# Non-volatile logic gates based on planar Hall effect in magnetic films with two in-plane easy axes

**DOI:** 10.1038/s41598-017-01219-z

**Published:** 2017-04-25

**Authors:** Sangyeop Lee, Seul-Ki Bac, Seonghoon Choi, Hakjoon Lee, Taehee Yoo, Sanghoon Lee, Xinyu Liu, M. Dobrowolska, Jacek K. Furdyna

**Affiliations:** 1 Physics Department, Korea University, Seoul, 136-701 Republic of Korea; 2 Physics Department, University of Notre Dame, Notre Dame, IN 46556 USA

## Abstract

We discuss the use of planar Hall effect (PHE) in a ferromagnetic GaMnAs film with two in-plane easy axes as a means for achieving novel logic functionalities. We show that the switching of magnetization between the easy axes in a GaMnAs film depends strongly on the magnitude of the current flowing through the film due to thermal effects that modify its magnetic anisotropy. Planar Hall resistance in a GaMnAs film with two in-plane easy axes shows well-defined maxima and minima that can serve as two binary logic states. By choosing appropriate magnitudes of the input current for the GaMnAs Hall device, magnetic logic functions can then be achieved. Specifically, non-volatile logic functionalities such as AND, OR, NAND, and NOR gates can be obtained in such a device by selecting appropriate initial conditions. These results, involving a simple PHE device, hold promise for realizing programmable logic elements in magnetic electronics.

## Introduction

Devices performing logic functions are key elements in processing information in electronic systems. Currently logic elements are primarily fabricated from semiconductor materials, in which their logic functionality is realized by utilizing the charge of the electron. Recently, however, magnetic devices have attracted a great deal of attention due to the advantages of non-volatility which these devices offer for information storage and processing. The best known such magnetic device is the magnetoresistive random access memory (MRAM), consisting of two ferromagnetic layers. Such MRAM element provides not only an efficient way of constructing memory devices, but also an opportunity to realize logic operations that can be reconfigured into AND, OR, NAND, or NOR gates by changing initial conditions^1, 2^. Owing to such flexibility for obtaining desired logic functionalities in a magnetically controlled system, various types of magnetic logic devices – such as domain wall logic, chameleon logic, and spin logic^3–8^ have been intensively investigated, with an eye on replacing current semiconductor-based logic devices.

In this context the vector characteristics of magnetization provide a new strategy for device design, based on the dependence of the device resistance on the direction of magnetization. For example, it is well known that the anisotropic magnetoresistance (AMR) of a magnetic film changes between maximum and minimum in a periodic fashion as the magnetization rotates in the film plane. Resistance values corresponding to stable magnetization orientations can then be utilized as logic states for storing information. This property of AMR has already been used for realizing a multi-state magnetic memory function by controlling domain structures in a single Hall device, and by manipulating the magnetization alignment in magnetic tunnel junctions^9–13^.

In the present study we exploit the phenomenon of the planar Hall resistance (PHR) exhibited by GaMnAs films^14^, and use its dependence on the direction of magnetization to achieve logic functionalities. The PHR in a GaMnAs film with a strong biaxial magnetic anisotropy is especially well suited for this purpose, since its value changes abruptly when the magnetization makes a transition between the magnetic easy axes in the film plane. By choosing an appropriate magnitude of the applied current as input signal, we are then able to demonstrate magnetic logic functions on a GaMnAs Hall device, as discussed later in this paper.

## Results and Discussion

**Figure 1:**
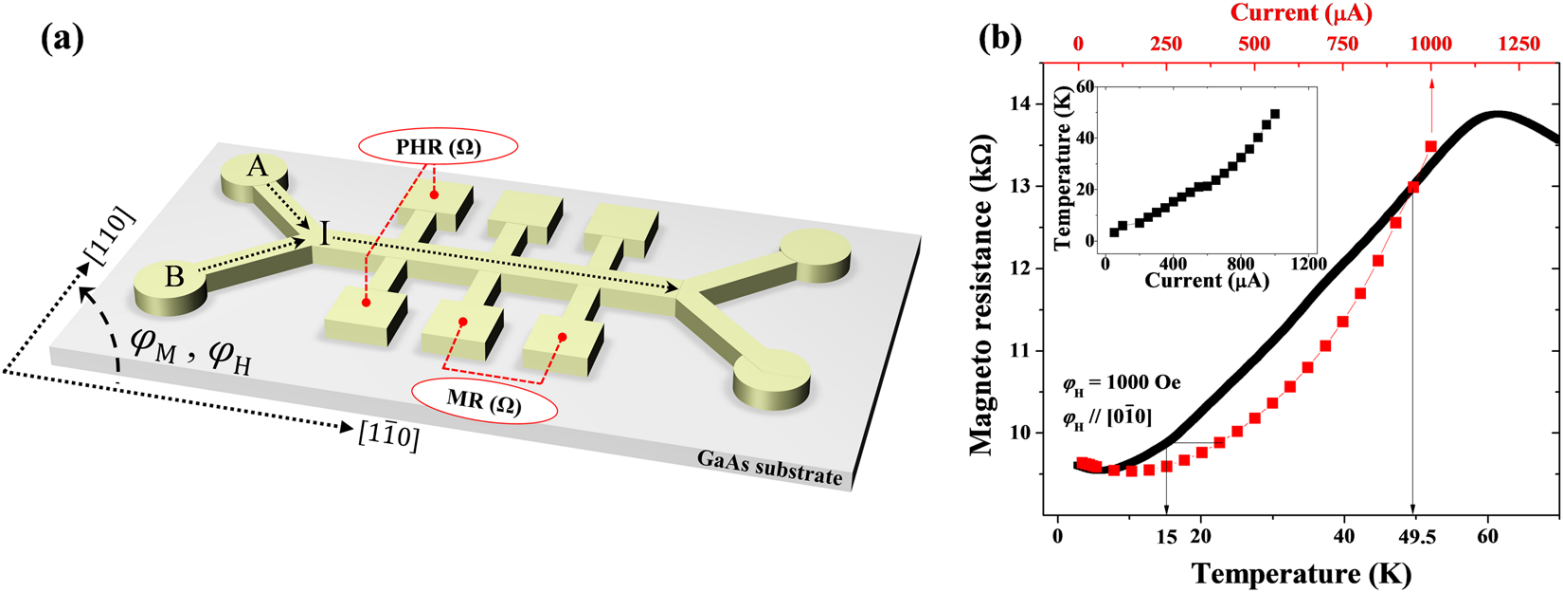
(**a**) Schematic diagram of a GaMnAs Hall device, showing transport measurement schemes and crystallographic directions. (**b**) Dependence of resistance on magnitude of current (upper scale) and on temperature (lower scale). Inset shows the dependence of device temperature on the current.

The magnetic logic function in our Hall device, shown schematically in Fig. 1(a), is based on the switching of magnetization between two nearly-orthogonal magnetic easy axes (i.e., between directions near the <100> crystal axes), at which maximum and minimum values of PHR occur^10, 14^. The process of these magnetization transitions in our Hall device must be carefully investigated at different current values, since the current flow during the measurement generates significant Joule heating, and thus affects the magnetic anisotropy of the GaMnAs layer, which is a sensitive function of temperature^15–19^. Since this feature will play a critical role in the present investigation, we carried out systematic measurements of such Joule heating effects in our device by changing the magnitude of the current over the range from 20 µA to 1 mA.

Figure 1(b) shows the dependence of resistance in our GaMnAs Hall device on the applied current and on temperature. All measurements were performed in the presence of a background magnetic field of 1000 Oe along the $[0\bar{1}0]$ direction. The application of such a strong magnetic field fixes the direction of magnetization in the direction of the applied field even when the magnetic anisotropy of the GaMnAs device changes with temperature. This process eliminates any resistance changes that might arise from relaxation of the magnetization due to the changes in magnetic anisotropy with changing temperature, which allows us to monitor only the dependence of the resistance on the temperature.

The black line in Fig. 1(b) represents the temperature dependence of the resistance in our Hall device measured with a current density of 20 µA, which is small enough not to generate any detectable Joule heating during the measurement. The temperature scan of the resistance in a 1000 Oe field shows a typical behavior of a GaMnAs layer, which exhibits a maximum resistance near the ferromagnetic transition temperature^15, 20^.

We then repeated the resistance measurement by systematically changing the magnitude of the sensing current while the temperature of the sample holder was set at 3K during the measurement. Resistances measured with different currents are plotted with red solid squares in Fig. 1(b). The plot shows that the resistance increases monotonically as the current increases from 20 µA to 1 mA. The value of the resistance measured at each current can be mapped into the resistance value obtained by scanning the temperature, as illustrated by thin horizontal lines and vertical arrows. Such mapping reveals the effective temperature in our Hall device at different values of the applied current, as plotted in the inset of Fig. 1(b). It is clear that the effective temperature of the device monotonically increases up to 50K as the current increases to 1 mA. This implies that the process of magnetization reversal in the device will be different at different values of the current due to differences in magnetic anisotropy of the GaMnAs layer induced by Joule heating.

Since the magnetic anisotropy of the GaMnAs layer is sensitive to the temperature^15, 19^, it is of critical importance for the present study to determine how the magnetic anisotropy in the GaMnAs layer – and thus of the orientation of its easy axes – depends on the applied current. We have investigated the magnetic anisotropy of our GaMnAs Hall device by measuring the angular dependence of the Hall resistance at several different currents, and have analyzed it in terms of the magnetic free energy given by^21, 22^

1 $$F=M[\frac{{H}_{C}}{8}{\cos }^{2}2{\phi }_{M}+\frac{{H}_{U}}{2}{\sin }^{2}{\phi }_{M}-H\,\cos ({\phi }_{M}-{\phi }_{M})]$$

where *H*_*C*_ is the cubic anisotropy field, *H*_*U*_ is the uniaxial anisotropy field associated with the $[1\bar{1}0]$ direction (i.e., the uniaxial hard axis), *φ*_*H*_ denotes the direction of the applied magnetic field, and *φ*_*H*_ is the direction of magnetization in the film. The value of PHR depends directly on the direction of magnetization, and thus its angular dependence observed by rotating the applied field can be fitted to the condition of free energy minimum by using the magnetic anisotropy fields as fitting parameters. This method of analysis is fully described in the literature^23, 24^ and we have adapted the same procedure to the present study (see Supplementary Information 1 for details), and have analyzed the angular dependence of the planar Hall resistance using four different currents. The resulting magnetic anisotropy fields are given in Table 1.

**Table 1:** Magnetic anisotropy values of GaMnAs layer measured at different currents.

Sensing current (µA)	Magnetic anisotropy field (Oe)	
	H_C_	H_U_
20	1106 ± 13	110 ± 6
100	966 ± 8	107 ± 4
250	724 ± 7	130 ± 3
400	408 ± 5	148 ± 2

**Figure 2:**
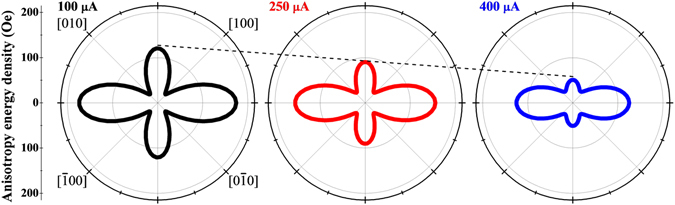
Magnetic free energy of GaMnAs layer obtained by using three different current values.

The free energy density diagrams corresponding to different current values can now be constructed using these anisotropy fields, as illustrated by Fig. 2. While the overall shape of the diagram showing the four energy minima is the same in all four cases, the uniaxial character of the anisotropy profile is significantly enhanced at higher currents. Of special importance in the present context is the reduction of the barriers at [110] and $[\bar{1}\bar{1}0]$, which will play a key role in allowing magnetization to switch between adjacent quadrants at specific values of the current, as discussed in later sections. These changes in the anisotropy of the GaMnAs layer illustrate the role of Joule heating by the current in the Hall device. Effects of such Joule heating in GaMnAs films had already been used for manipulating magnetization in GaMnAs-based magnetic tunnel junctions^25^. Here we will use such current-induced thermal effects to achieve logic operations. Note that changes in anisotropy of the GaMnAs layer (which allow the magnetization to reorient itself in the GaMnAs layer, and result in the intended logic operation) occur only while the current pulse is applied. As soon as the pulse ends (and the logic operation is completed), the material returns to its original predominantly cubic anisotropy.

**Figure 3:**
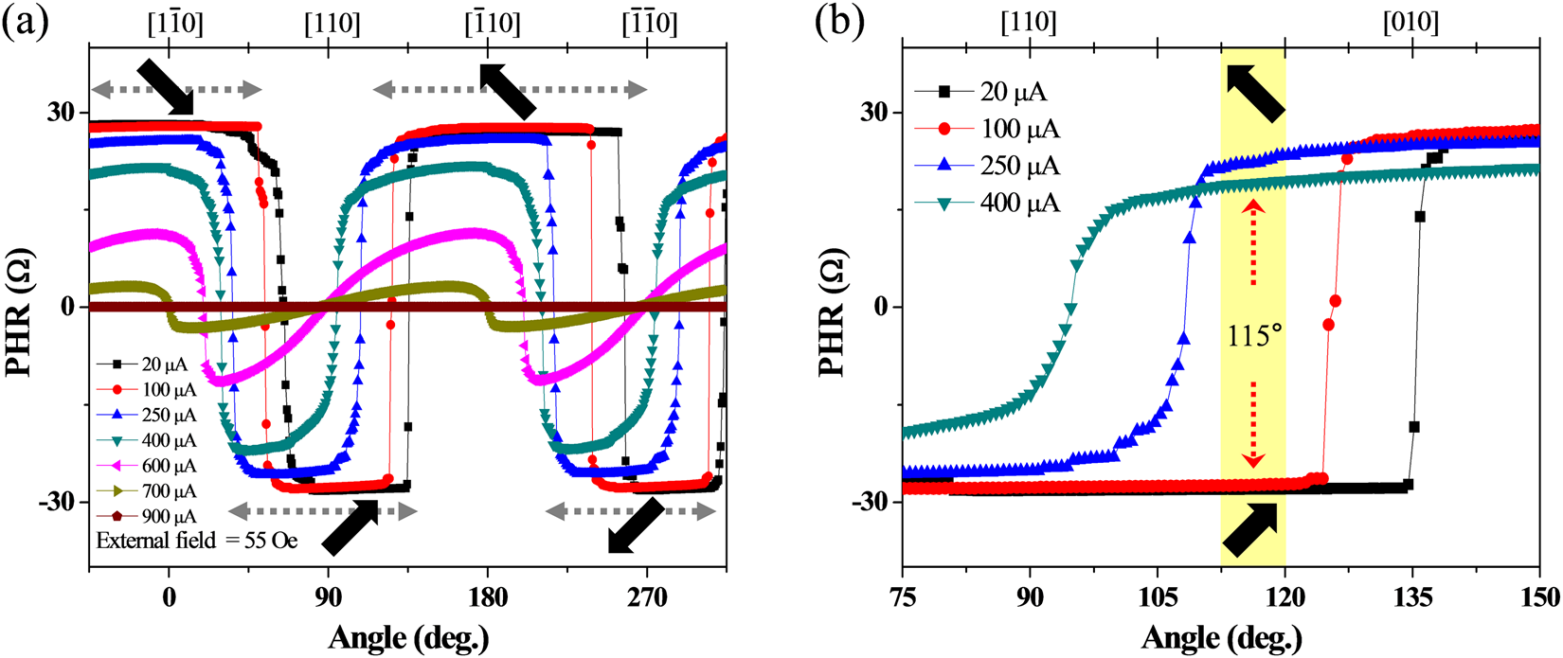
(**a**) Dependence of PHR on field orientation observed at various currents, from 20 µA to 900 µA, as an external field of 55 Oe is rotated CCW over 360°. Black thick arrows show the direction of magnetization at different field orientations during magnetization reversal. (**b**) Expanded view of the region between 75° and 150°. Importantly, the direction of magnetization can be different at the same field orientation for different currents, as illustrated by the behaviour in the yellow shaded region.

We now monitor the process of magnetization reversal in the Hall device at different currents. Figure 3(a) shows PHR data obtained at several current values between 20 µA and 900 µA, observed as an external field of 55 Oe is rotated counterclockwise (CCW) over 360°. The thick black arrows show the directions of magnetization at specific field orientations during the reversal process. As the current increases, the PHR clearly shows a reduction of its amplitude, a weakening of the abruptness of the transition, and (importantly) a shift of the field angle at which the transition occurs. These changes in the PHR observed with increasing current are very similar to those typically observed with increasing sample temperature, which causes the magnetization of GaMnAs layer to decrease, and its magnetic anisotropy to change^15, 18, 19^.

Because of the shift in the field angle at which the transition of magnetization takes place at different currents, one can find specific field orientations at which the direction of magnetization in the device is different for different current values. To elucidate this, in Fig. 3(b) we plot the PHR measured with four different currents for field orientations between 75° and 150°. When the external field direction is 115° (see position marked with red dotted arrows), PHR shows minima for currents of 20 and 100 µA, and maxima for 250 and 400 µA. This indicates that PHR can be switched between minimum and maximum by changing the magnitude of the current at a fixed magnetic field direction. Such tunability of PHR by the current in the Hall device can thus be used to realize magnetic logic functions, as discussed below.

## Logic operations with Hall device

We now discuss the application of our Hall device as a logic gate. Typically, logic operations involve two input values (such as input currents into terminals A and B in the device in Fig. 1), which are converted by the device to one specific output. In binary logic, the inputs and outputs are assigned the values “0” and “1”^2, 26^. Consistent with Fig. 3(b), we have chosen two values of the current, 50 µA and 200 µA, as low and high input values, respectively. We use these values to represent the “0” and “1” input logic states in order to illustrate the logic operation of our Hall device. In this case the output is detected by the PHR of the device, with its minimum and maximum values representing the “0” and the “1” output logic states, respectively. The description for input and output signals and corresponding logic states are summarized in Table 2. Note, however, that the PHR value (high or low) depends only on the orientation of magnetization, as seen in Fig. 3(b) near 115°, and not on the current amplitude. We can thus use a current pulse to change the orientation of the magnetization (and thus to change the value of PHR), which will then remain in its new state after the pulse is removed.

**Table 2:** Definition for INPUT and OUTPUT signals.

INPUT		OUTPUT	
States	Current (µA)	States	PHR (Ω)
Low (0)	50	Low (0)	−26
High (1)	200	High (1)	+26

### The AND logic function

**Figure 4:**
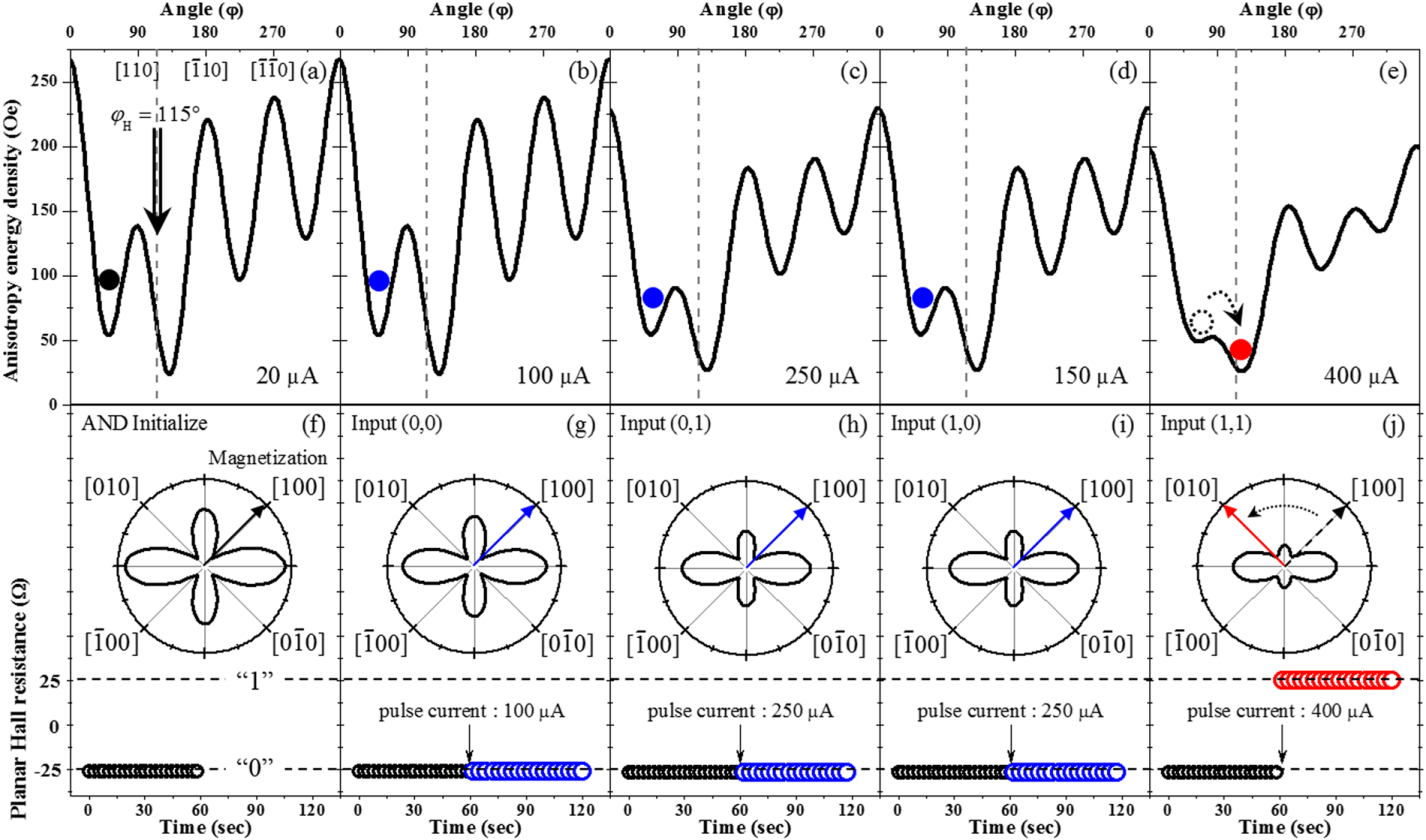
Magnetic free energy profiles for the initial state (**a**) and during application of 0.1 s current pulse with magnitudes of 100, 250, 250, and 400 µA (**b–e**). Dotted vertical lines in panels (a–e) indicate the orientation of background field, and solid circles indicate directions of magnetization. Orientation of magnetization is also shown in polar plots (**f–j**). Dotted and solid arrows indicate directions of magnetization before and after current pulses. PHR values measured 60 s before and after current pulses are shown in lower part of the panels (f–j).

The AND logic function is realized when the two inputs into terminals A and B of our device together result in an output signal as defined by Table 3. To test the AND logic function in our Hall device, we set the device using the following procedure. The magnetization of the device is first saturated by a strong field of 2000 Oe applied in the fourth quadrant, along −60°. The field is then reduced to 55 Oe (referred as background field in what follows) and its direction is rotated CCW to the 2^nd^ quadrant, to 115°. Importantly, the value of 55 Oe for the background field used in this initialization is insufficient to complete the full magnetization reversal, and the magnetization of the device is only transferred to the free energy minimum position in the first quadrant during the CCW rotation of the field, as shown in Fig. 4(a) and (f). Although the energy minimum is deeper in the second quadrant than in the first due to the presence of the background field of 55 Oe along the 115° orientation, the barrier between the two minima is sufficiently high to keep the magnetization at the 1^st^ quadrant minimum. This initial setting of magnetization results in the low PHR value of −26 Ω, as plotted with black open circles (see the bottom part of Fig. 4(f)), which corresponds to the output logic state “0”.

**Table 3:** AND Logic.

INPUT			OUTPUT	
A	B	Pulse current (µA)	PHR(Ω)	State
0	0	100	−26	0
0	1	250	−26	0
1	0	250	−26	0
1	1	400	+26	1

We now test the AND logic function given in Table 3 by applying four different combinations of input current pulses to the device in Fig. 1. Figure 4(b–e) shows the magnetic free energy profiles of the GaMnAs layer for the four current pulses of 0.1 s duration with input combinations (0,0), (0,1), (1,0), and (1,1) (where, as mentioned earlier, “0” stands for 50 µA and “1” for 200 µA at terminal A or B of the device), resulting in total currents of 100, 250, 250, and 400 µA, which correspond to current densities of 1 × 10^4^, 2.5 × 10^4^, 2.5 × 10^4^, and 4 × 10^4^ A/cm^2^, respectively. Note that these current densities are smaller than those used in most magnetic devices, where current densities in the range of 10^5^ to 10^7^ A/cm^2^ are normally used for manipulating magnetization^27, 28^. Since the magnetic anisotropy values of our Hall device measured with currents of 100, 250, and 400 µA were already obtained in the study of the angular dependence of PHR (see Table 1 and Fig. 2), we use these values to plot the energy diagrams corresponding to the pulses. Here the solid circles indicate the directions of magnetization in a given diagram, and the open dotted circle represents the direction of magnetization before it makes a transition caused by the current pulse. The transition of the magnetization between the two energy minima depends on both the energy difference of the minima and the barrier height between them^29–32^. For the 100 and 250 µA current pulses (i.e., (0,0), (0,1), and (1,0) input combinations), the magnetic anisotropy change in the GaMnAs layer due to Joule heating is insufficient for a transition to occur between the two energy minima, so that the magnetization remains at the initial position, as shown in Fig. 4(b–d). Thus the PHR value also remains at its initial minimum (i.e., at the “0” logic state) seen in Fig. 4(g–i), where the directions of the magnetization are shown as blue arrows in the polar plots. The PHR values measured for 60 s before and after the pulse are plotted at the bottom part of each panel as black and blue open circles, respectively.

However, for the current pulse with 400 µA (i.e., the (1,1) input combination), the modification of the magnetic anisotropy in the device is sufficient to cause the transition of magnetization from its initial orientation in the 1^st^ quadrant to the energy minimum in the second quadrant, as shown in Fig. 4(e) (see Supplementary Information 2 for additional details). This magnetization transition causes the PHR to jump to its high value (i.e., to “1” logic state), as shown in Fig. 4(j), where the direction of the magnetization and the resulting PHR value are shown as a red arrow and red open circles. The jump occurs as a single step between two states, in the form of two consecutive PHR values measured in 500 ms intervals before and after the current pulse. The PHR after the pulse remains constant at its new value, as seen in Fig. 4(j), indicating that the transition of the entire magnetization is completed within 500 ms. Furthermore, the PHR in our experiment changed only between two values, +26 Ω and −26 Ω, that correspond to the logic state “1” and “0”. We do not observe any other stable PHR values during the operation of the logic function, clearly indicating a single-domain behavior of our device. Indeed, the domain size of GaMnAs film is known to be of the order of hundreds of micro-meters^33^, which is comparable to our device size. This indicates that the logic operation in our device can be understood in terms of single-domain structures. The PHR output responses of the device to these input current combinations correspond exactly to the AND logic operation given in Table 3, indicating that the Hall device functions as an AND logic element.

### OR logic functions

**Figure 5:**
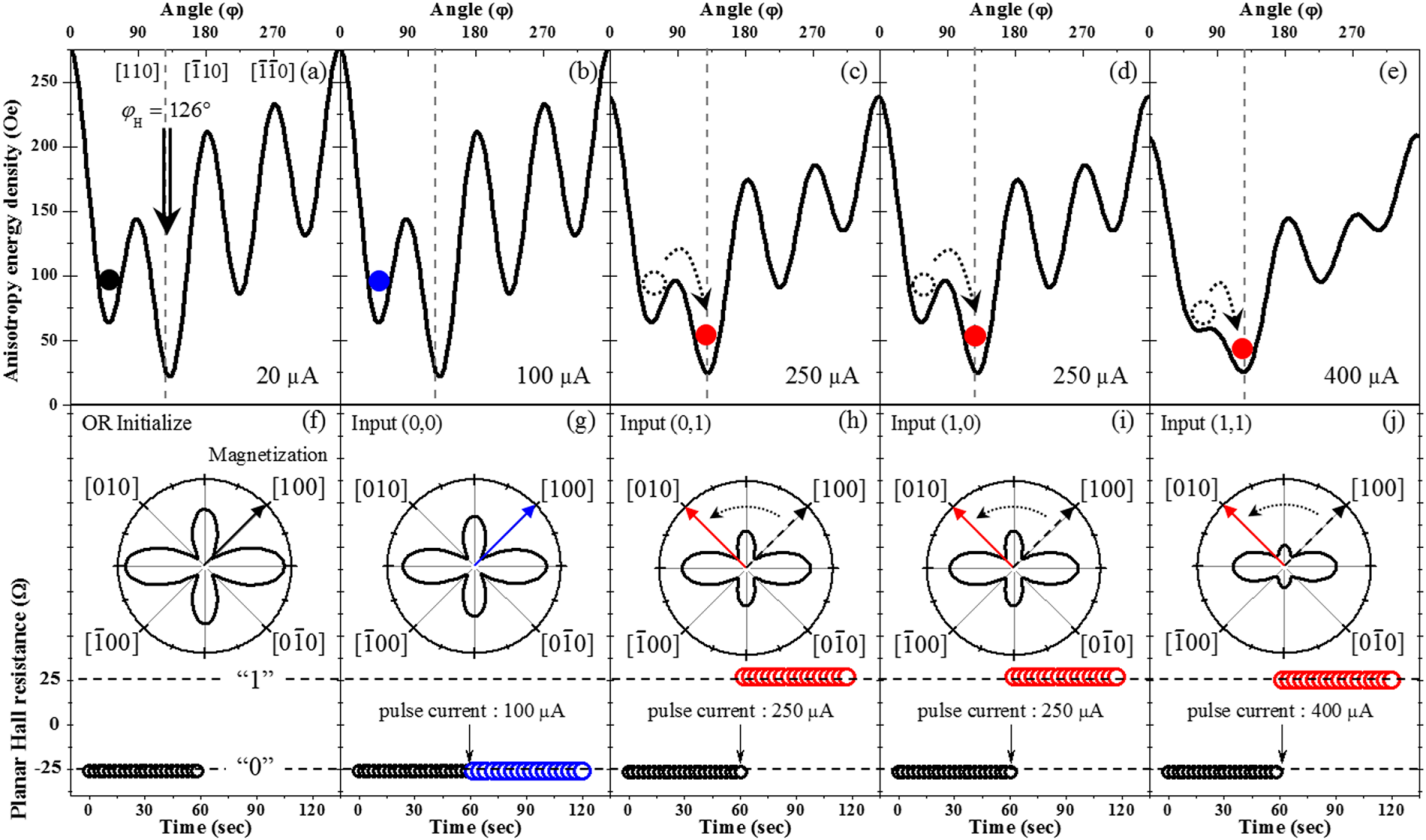
Magnetic free energy profiles for the initial state (**a**) and during application of 0.1 s current pulses of 100, 250, 250, and 400 µA (**b**–**e**). Dotted vertical lines in panels (a–e) indicate the direction of the background field, and solid circles show the direction of the magnetization. Directions of magnetization are also shown in the polar plots (**f–j**), where dotted and solid arrows indicate directions of the magnetization before and after current pulses, respectively. PHR values measured 60 s before and after the current pulses are shown at the bottom of the panels (f–j).

We now consider the operation of the Hall device as an OR gate, represented by Table 4. For this function, we follow the same initialization process as that used for the AND logic, but after reducing the field strength to 55 Oe, this field is now rotated to 126° (see dotted vertical line in Fig. 5). Figure 5(a) shows the magnetic free energy profile for the initial state. The magnetization of the device still lies along the free energy minimum position in the 1^st^ quadrant, as shown in Fig. 5(a) and (f). However, the transition of magnetization will be different from the AND logic operation, because the background field direction is now closer to the magnetic easy axis in the 2^nd^ quadrant (i.e., it is oriented at 126°, as compared to 115° for the AND logic). This initial condition makes the energy minimum in the 2^nd^ quadrant deeper than was the case for the AND function, making the transition of magnetization to the second quadrant easier.

**Table 4:** OR Logic.

INPUT			OUTPUT	
A	B	Pulse current (µA)	PHR(Ω)	State
0	0	100	−26	0
0	1	250	+26	1
1	0	250	+26	1
1	1	400	+26	1

Figure 5(b–e) show the magnetic free energy profiles of the GaMnAs layer after applying 0.1 s current pulses of 100, 250, 250, and 400 µA, which represent the four input combinations of (0,0), (0,1), (1,0), and (1,1), respectively. For the current pulses with 100 µA, the magnetic anisotropy change in the GaMnAs layer due to the impulse of Joule heat does not result in sufficient difference between the energy profiles in the 1^st^ and 2^nd^ quadrants to enable the magnetization to switch from its initial position, as shown in Fig. 5(b). The output logic state thus remains at “0” for this set of inputs. However, as can be seen from Fig. 5(c–e), for the 250 and the 400 µA pulses anisotropy changes (energy minimum in the 2^nd^ quadrant and barrier height between the quadrants) becomes sufficient to enable the magnetization transition to the minimum the 2^nd^ quadrant. This transition is shown by the black dotted and red solid circles in Fig. 5(c–e). The magnetization directions after the transition are shown in the corresponding bottom panels, where red arrows indicate the direction of the magnetizations after the transition. The PHR values measured for a period of 60 s before and after each input pulse are shown in the bottom part of Fig. 5(g–j). Thus after the (0, 1), (1, 0) and (1, 1) inputs, the PHR signal clearly jumps from −26 Ω to +26 Ω, leading to a change of the output logic state from “0” to “1”. Thus the observed output response to the input in the device now follows the OR logic, as given in Table 4.

The device can also be operated after the magnetization is initialized by orienting the magnetization at the energy minimum in the second quadrant, which results in the high value of PHR, corresponding to the output logic state “1”. With this initial state, the device can act as a NAND or a NOR gate, depending on the selected direction of the background field. Detailed discussion of the NAND and NOR logic operations is given in Supplementary Information 3.

Magnetic logic operation based on the Hall effect has been reported in several articles^34–36^. In particular, Zhan *et al*.^37^, reported a similar type of PHE magnetic logic device consisting of a piezoelectric transducer and a ferromagnetic Co_2_FeAl layer^37^. The Co_2_FeAl layer has a biaxial magnetic anisotropy in the film plane, that results in two orthogonal in-plane magnetic easy axes, just as in our GaMnAs layer. The magnetic anisotropy of the film in ref. 30 is controlled by the piezoelectric voltage to achieve the transition of the magnetization between the easy axes. The NOR gate function is achieved in this case by combining two Hall devices, each of which is fabricated along one of the two orthogonal crystal directions (i.e., [100] and [010]). In contrast, our PHE logic device does not involve any other materials system to manipulate the magnetic anisotropy of the magnetic film. The modification of magnetic anisotropy in our device is achieved by the input current pulse through Joule heating, and the device structure required for performing logic functions is fabricated from a single magnetic layer having two in-plane magnetic easy axes, such as the GaMnAs film used in this study. Furthermore, logic functions performed by the device can be altered by appropriate initializing processes, as demonstrated above. The monolithic structure of the device, along with its reconfigurable property that allows to alter the logic functions which it performs, not only offers new options for magnetic logic operations, but also provides more flexibility for designing compact electronic circuits for practical device applications.

In conclusion, we have investigated the magnetic logic functionality of a GaMnAs Hall device based on the planar Hall effect. The logic operations are achieved through the dependence of the magnetic anisotropy of the device on the applied current. By appropriately choosing the input currents, and detecting PHR as the output, AND, OR, NAND, or NOR magnetic logic functions were achieved using a single-element Hall device simply by setting appropriate initial conditions. For purposes of this study the logic functions are demonstrated by using a global background field. The function of this field can, however, be replaced by other effects, such as exchange bias^38, 39^ or current-induced spin-orbit fields^28, 40, 41^, as required by practical requirements. The logic operations discussed in this paper, although achieved at low temperatures, provide important opportunities for investigating “proof-of-concept” device structures in which various logic strategies can be effectively explored.

## Methods

Ferromagnetic GaMnAs layers used in this study were grown by molecular beam epitaxy on (001) GaAs substrates. Prior to the growth of the GaMnAs layer, a 100 nm GaAs buffer layer was deposited on the substrate at 600 °C, followed by deposition of a 2 nm GaAs buffer at 250 °C. A 100 nm Ga_1−*x*_Mn_*x*_As film with *x* = 0.08 was then grown on top of the GaAs buffer. For investigating the logic functionality based on the planar Hall effect, we patterned a Hall device on the GaMnAs film by photolithography and chemical wet etching, shown schematically in Fig. 1(a). The Hall bar was in the form of a strip 200 µm long and 10 µm wide, with the long dimension aligned with the $[1\bar{1}0]$ direction of the GaAs substrate. Even though the width of the Hall device used here is 10 μm, it can be significantly scaled down to nanometer scale, as long as the magnetic anisotropy remains well defined in the device. Magnetoresistance and Hall resistance measurements were used to investigate the Curie temperature, Joule heating, magnetic anisotropy, and possible logic operations of the Hall device. In the experiments described in this paper, orientations of the applied magnetic field *φ*_*H*_ and of magnetization of the GaMnAs film *φ*_*M*_ are measured counterclockwise (CCW) from the $[1\bar{1}0]$ crystallographic direction (i.e., from the direction of the current flow) in the (001) crystal plane, as shown in the Fig. 1(a).

## Electronic supplementary material

Supplementary Material - Non-volatile logic gates based on planar Hall effect in magnetic films with two in-plane easy axes
