# Supplementary material for: Non-volatile logic gates based on planar Hall effect in magnetic films with two in-plane easy axes
Source: Sci Rep. 2017 Apr 25;7:1115. doi: 10.1038/s41598-017-01219-z (PMC5430745; doi:10.1038/s41598-017-01219-z)
Supplement: Supplementary file 1 — Supplementary Material - Non-volatile logic gates based on planar Hall effect in magnetic films with two in-plane easy axes [file 41598_2017_1219_MOESM1_ESM.doc]

**Non-volatile logic gates based on planar Hall effect in magnetic films with two in-plane easy axes**

**- Supplementary Material -**

Sangyeop Lee, Seul-Ki Bac, Seonghoon Choi, Hakjoon Lee, Taehee Yoo, and Sanghoon Lee*

*Physics Department, Korea University, Seoul 136-701, Republic of Korea*

Xinyu Liu, M. Dobrowolska, and Jacek K. Furdyna

*Physics Department, University of Notre Dame, Notre Dame, IN 46556, USA*

**Supplementary information 1**

Magnetic anisotropy of a ferromagnetic film can be obtained by measuring the angular dependence of the planar Hall resistance (PHR) and analyzing the results in terms of magnetic free energy based on the Stoner-Wohlfarth model[^1^](#_ENREF_1). In the case of a ferromagnetic films with in-plane magnetic easy axes, such as a GaMnAs film grown on a (001) GaAs substrate studied in the present paper, information on the direction of magnetization in the film plane is contained in following equation for PHR:[^2^](#_ENREF_2)

, (S1)

where *k* is a constant related to the anisotropic magnetoresistance; *M* is the magnetization of the film; *t* is its thickness; and is the angle between the projection of the magnetization on the (001) plane and the $[1\bar{1}0]$ crystallographic direction.

The magnetic free energy, which determines the direction of magnetization for any given orientation of the applied field, can be written as[^3^](#_ENREF_3)

, (S2)

where $H_{C}$ is the in-plane four-fold symmetric cubic anisotropy field, $H_{U}$ is the in-plane uniaxial anisotropy field, gives the directions of the magnetization, and the direction of external field in the film plane. The two equations, S1 and S2, are used to analyze the angular dependence of PHR.


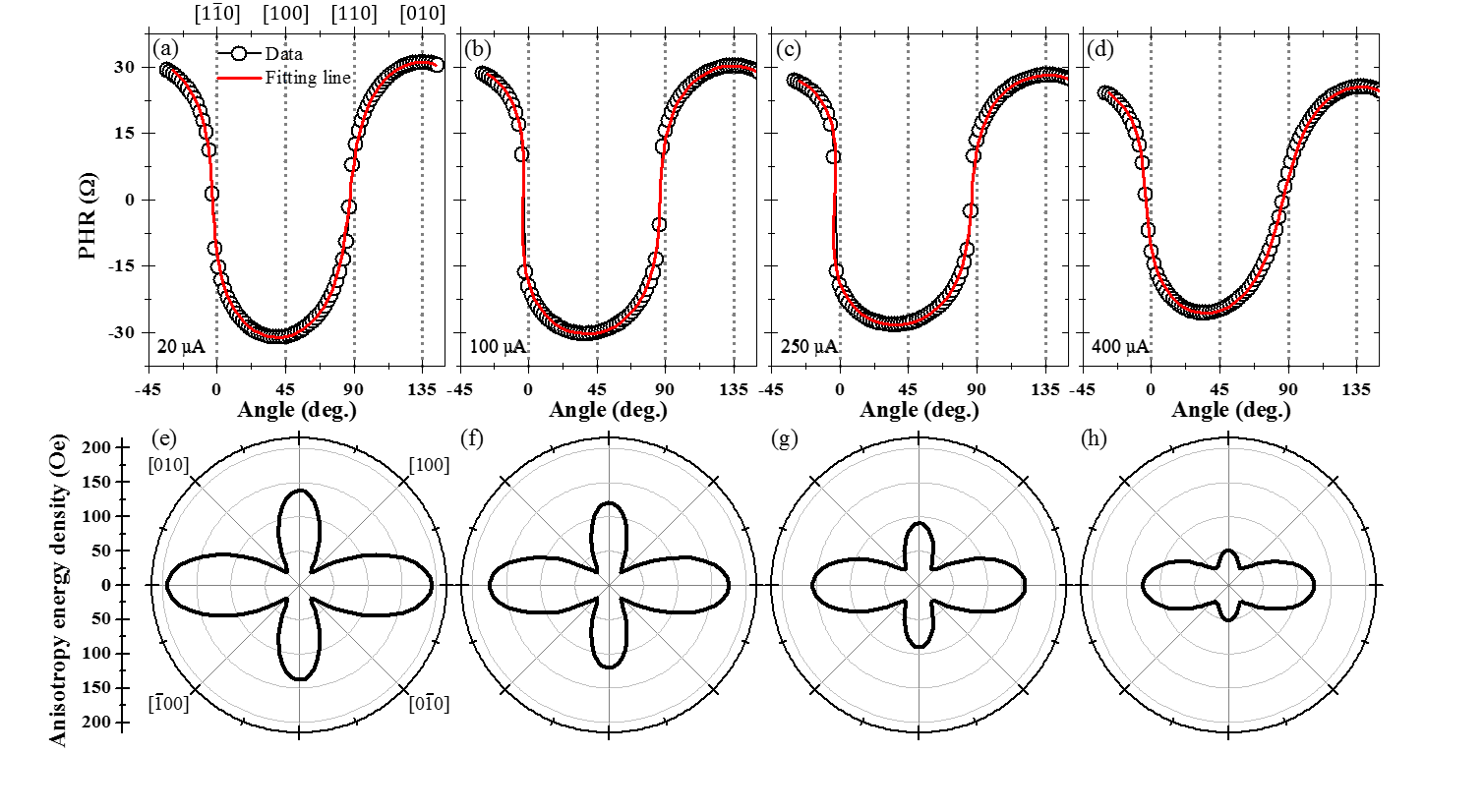


Fig. S1. (upper panels) Planar Hall resistance data obtained with four different currents by rotating the applied field in the (001) plane of the GaMnAs layer (open circles); and corresponding theoretical fit obtained using Eqs. (S1) and (S2) are shown with solid lines. (bottom panels) Magnetic free energy diagrams corresponding to panels in top row.

The angular dependence of PHR measured at 3 K at four different current values is shown as open circles in the top panels of Fig. S1. Changes in PHR with increasing current in the GaMnAs film are very similar to those observed with increasing temperature.[^4^](#_ENREF_4) This indicates that the current flowing through the film generates Joule heating during the measurement, changing the local temperature in the device. Since the direction of magnetization in the GaMnAs film follows the magnetic free energy minimum as the applied magnetic field is rotated, the data plotted in Fig. S1 can be fitted using the conditions for free energy minimum (i.e., and ) with magnetic anisotropy fields $H_{C}$and $H_{U}$ as fitting parameters.[^5^](#_ENREF_5) The solid curves in the top panels of Fig. S1 represent best fits obtained by this fitting process. The magnetic anisotropy fields obtained by this fitting process for four current values are shown in Table S1. Free energy diagrams constructed by using these anisotropy fields are plotted in the bottom row of Fig S1. While the overall shape of free energy diagrams are qualitatively similar, showing four energy minima in all four cases, the magnitudes of the energy barriers are significantly reduced for larger currents, indicating the significance of Joule heating by the current in our Hall device. Note in particular the reduction of the energy barriers along [110] and $[\bar{1}\bar{1}0]$ directions, a feature that plays a key role in enabling the switching of magnetization between adjacent quadrants as current pulse is applied.

Table. S1. Magnetic anisotropy parameters

| Sensing  current (µA) | Magnetic anisotropy field (Oe) | |
| --- | --- | --- |
|  | **H_C_** | **H_U_** |
| 20 | 1106 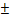 13 | 110 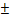 6 |
| 100 | 966 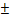 8 | 107 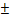 4 |
| 250 | 724 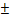 7 | 130 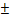 3 |
| 400 | 408 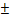 5 | 148 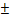 2 |

**References**

1. Stoner EC, Wohlfarth EP. A mechanism of magnetic hysteresis in heterogeneous alloys. *Philos Trans R Soc London, Ser A* **240**, 599-642 (1948).

2. Okamoto K. A new method for analysis of magnetic anisotropy in films using the spontaneous Hall effect. *J Magn Magn Mater* **35**, 353-355 (1983).

3. Liu X*, et al.* Perpendicular magnetization reversal, magnetic anisotropy, multistep spin switching, and domain nucleation and expansion in Ga1-xMnxAs films. *J Appl Phys* **98**, 063904 (2005).

4. Shin DY, Chung SJ, Lee S, Liu X, Furdyna JK. Temperature dependence of magnetic anisotropy in ferromagnetic (Ga,Mn)As films: Investigation by the planar Hall effect. *Phys Rev B* **76**, 035327 (2007).

5. Son H, Chung SJ, Yea SY, Lee S, Liu X, Furdyna JK. Quantitative investigation of the magnetic anisotropy in GaMnAs film by using Hall measurement. *J Appl Phys* **103**, 07F313 (2008).

**Supplementary information 2**

Here we explain how the application of a current pulse causes a transition of magnetization between energy minima in our GaMnAs Hall device. As a representative case, we describe the magnetization transition caused by the application of a 400 µA current pulse (i.e., a (1,1) input combination) to an AND gate. Figure S2 shows the magnetic free energy profiles of the GaMnAs layer before, during, and after the 400 µA current pulse. At the initial state, the energy minimum is deeper in the second quadrant than in the first due to the presence of the background field of 55 Oe in the 115° orientation, as shown in the top left panel of the figure. However, the barrier between the two minima is sufficiently high to keep the magnetization at the 1^st^ quadrant minimum, as shown by the solid dot, and by the solid arrow in the polar plot. This initial setting corresponds to the low PHR value of -26 Ω, plotted with black open circles (see bottom panel of Fig. S2), which corresponds to the output logic state “0”, and remains in that state until the current pulse arrives.

However, the magnetic free energy profile of the GaMnAs layer changes significantly during the current pulse due to Joule heating, which reduces the magnetic anisotropy energy. This is shown in Cartesian and in polar coordinates in the middle panels, top and middle row. Note that the barrier between the two energy minima is lowered during the pulse, enabling the magnetization to make the transition to the deeper minimum in the second quadrant. This reorientation of the magnetization causes the PHR value to change to 26 Ω, as plotted with red open circles (bottom panel of Fig. S2), which corresponds to the output logic state “1”. After the current pulse, the magnetic energy profile returns to its original state, but the direction of magnetization is now along the minimum in the second quadrant. Since this energy minimum is the deepest in the energy profile, the magnetization remains in that position as a stable state, as indicated by the PHR value of 26 Ω shown in the figure.


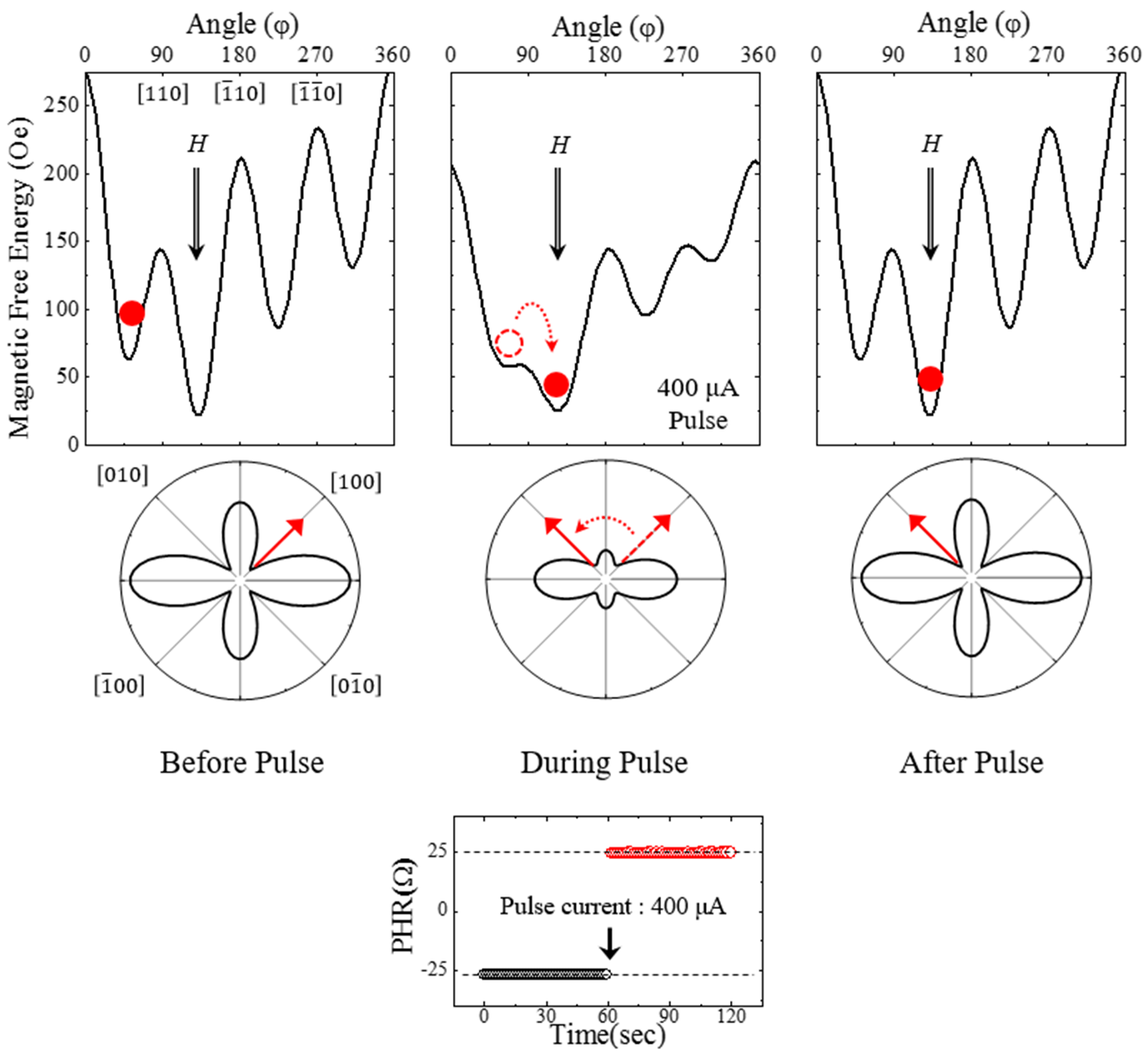


Fig. S2. Magnetic free energy profile for the initial state before the pulse (left), during the 0.1 s current pulse of 400 µA (middle), and after the pulse (right). Downward arrows indicate the direction of the applied field, and solid circles show the directions of magnetization. The orientation of magnetization before and after the current pulse is also shown in the polar plots (second row). The PHR values measured for 60 s before and after the pulse are shown in the bottom panel.

**Supplementary information 3**

***NAND logic function***:

The NAND logic function is realized by the negation of AND function. The output is low if both inputs into terminals A and B are high. Otherwise it is high, as shown in Table S2. The device can be used after the initialization of the magnetization along the easy axis in the second quadrant, which gives a high PHR state corresponding to the logic state “1”. For this initialization, we again apply a strong field of 2000 Oe along 240° to saturate the magnetization of the device. The field strength is then reduced to 55 Oe and its direction is rotated CW to 60°. This process initializes the magnetization of the device along the free energy minimum in the second quadrant, as shown in Fig. S3(a) and (f). At this initialized condition, we repeatedly test the NAND logic process given in Table S2 by applying 0.1 s current pulses with magnitudes of 100, 250, 250, and 400 µA, corresponding to the two input pulse combinations of (0,0), (0,1), (1,0), and (1,1) to the A and B terminals of the Hall device.

Figures S3(a) – S3(e) shows the magnetic free energy profiles of the GaMnAs layer before (a) and after application of the 0.1 s current pulses with magnitudes of 100, 250, 250, and 400 µA, which represent four input combinations of (0,0), (0,1), (1,0), and (1,1), respectively. The magnetization directions in the polar plot and corresponding output voltages measured after each input pulse are shown in Fig. S3(g)-S3(j). For current pulses with 100 and 250 µA (i.e., (0,0), (0,1), and (1,0) combination of inputs) the magnetization cannot make the transition from 2^nd^ to 1^st^ quadrant, and thus the PHR output value remains at its initial maximum (i.e., at logic state “1”). However, the application of the current pulse with 400 µA (i.e., (1,1) combination of input) makes the magnetization transition to the energy minimum in the first quadrant (see Fig. S3(j)) and the PHR jumps to its minimum value (i.e., “0” logic state). These output responses to the input combinations in the device follows the NAND logic function given in Table S2.


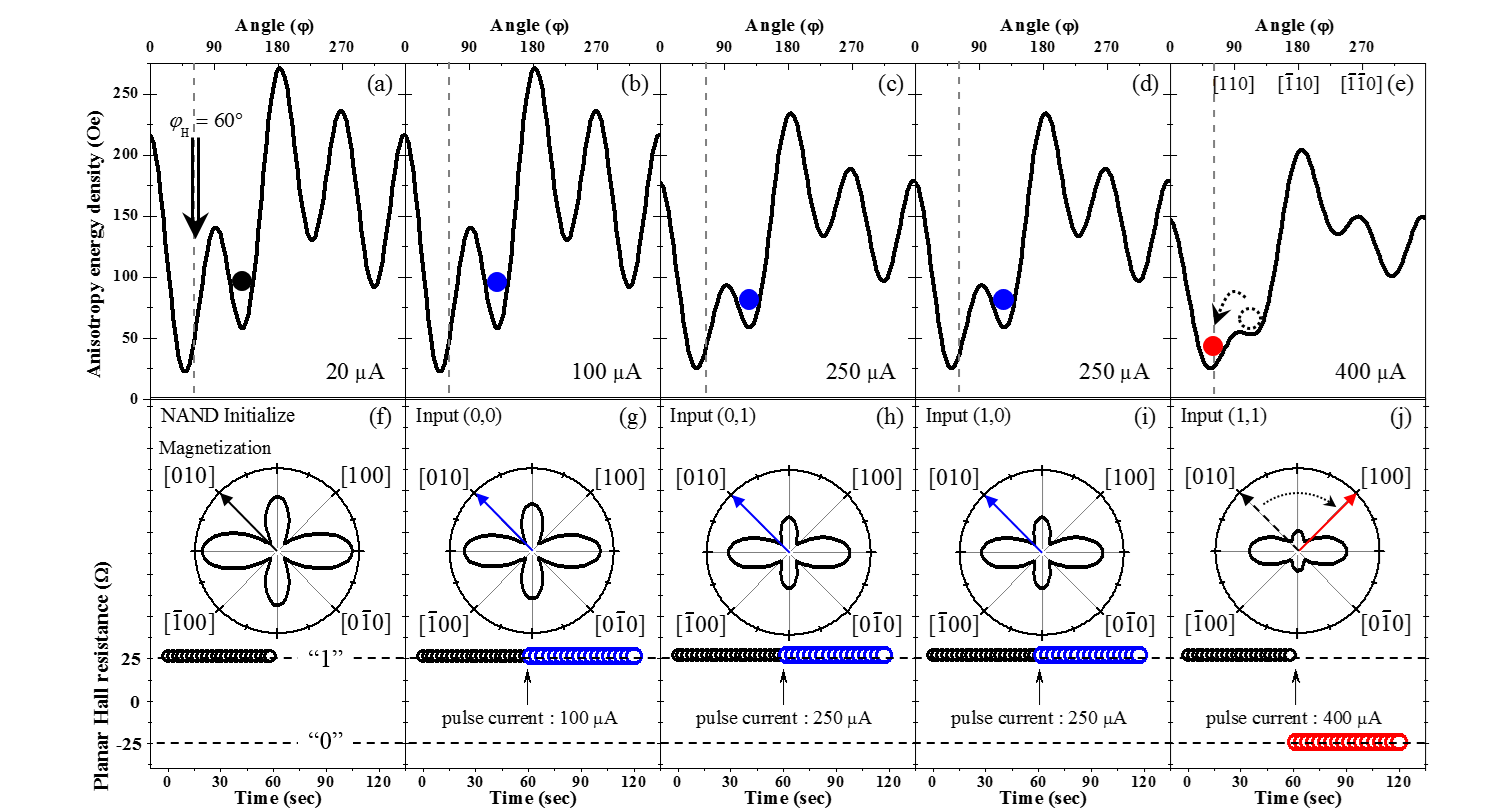


Fig. S3. Magnetic free energy profile for initial state (a), and during application of 0.1 s current pulses with magnitudes of 100, 250, 250, and 400 µA (b)-(e). Dotted vertical line indicates the direction of the background field, and solid circles indicate the orientation of magnetization. Direction of magnetization shown in the polar plot (f)-(j). Dotted and solid arrows indicate the directions of the magnetization before and after current pulses. The PHR values measured 60 s before and after current pulses are shown in the bottom part of panels (f)-(j).

Table. S2. NAND Logic

| INPUT | | | OUTPUT | |
| --- | --- | --- | --- | --- |
| A | B | Pulse current (µA) | PHR(Ω) | State |
| 0 | 0 | 100 | +26 | 1 |
| 0 | 1 | 250 | +26 | 1 |
| 1 | 0 | 250 | +26 | 1 |
| 1 | 1 | 400 | -26 | 0 |

***NOR logic functions***:

The NOR logic function is realized by the negation of the OR function. The output is high if both inputs into terminals A and B are low. Otherwise the output is low as shown in Table S3. The magnetization of the Hall device is again set to the energy minimum position in the second quadrant following the same procedure as that used for the NAND logic operation. Now, however, the 55 Oe background field is rotated to 45°, i.e., closer to the easy axis in the first quadrant. Figure S3(a) shows the magnetic free energy profile for the initial state. Even with this initialization, the magnetization of the device lies along the free energy minimum position in the second quadrant as shown in Figs. S4(a) and S4(f). However, now the process of magnetization transition will be different from that of the NAND case, because the background field is oriented closer to the easy axis (i.e., it is now at 45°, compared to 60° used for NAND). This initial condition makes the energy minimum in the first quadrant deeper than was the case for NAND, making the transition of magnetization to the first quadrant easier.

We repeated the application of the 0.1 s current pulses with four different currents (i.e., 100, 250, 250, and 400 µA) to the Hall device, and measured the PHR to test the performance of the Hall device executing the NOR logic function. Figures S4(b) – S4(e) show the magnetic free energy profiles of the GaMnAs layer after the application of 0.1 s current pulses with magnitudes 100, 250, 250, and 400 µA. For the 100 µA current pulse, the magnetic anisotropy change in the GaMnAs layer does not make the barrier between the energy minima in the 2^nd^ and 1^st^ quadrants sufficiently low, so that the magnetization remains at its initial position, as shown in Fig. S4(b). Therefore, the output logic state is kept at the logic state “1”. However, as can be seen from Fig. S4(c)–(e), the barrier height of becomes low enough compared to the difference between energy minima in the 2^nd^ and 1^st^ quadrants to make the transition of the magnetization to the minimum in the 2^nd^ quadrant for the 250 and 400 µA pulses. The resulting directions of magnetization after these transitions are shown in the corresponding bottom panels, where red arrows in the polar plots indicate the directions of the magnetization after pulse-induced transitions. The PHR values measured for 60 s before and after each input pulse are shown in the bottom part of Fig. S4, panels (g) – (j). The PHR signal clearly jumps from +26 Ω to -26 Ω after the input pulse, leading to the change of the output logic state from “1” to “0”. The observed output responses to the inputs in the device represent exactly the NOR logic function given in Table S3.


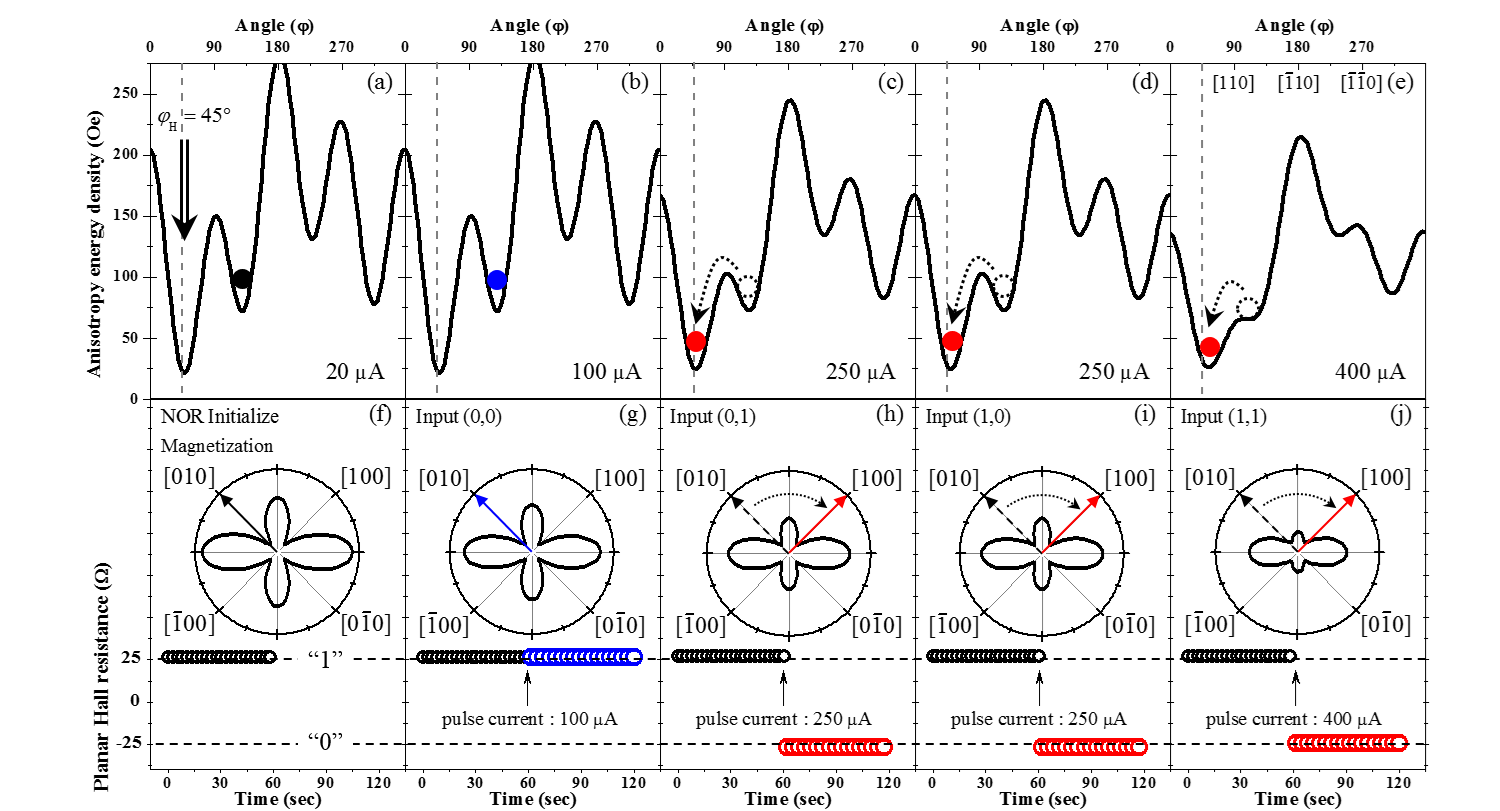


Fig. S4. Magnetic free energy profile for the initialized state (a) and during application of 0.1 s current pulse with magnitudes of 100, 250, 250, and 400 µA (b)-(e). Dotted vertical lines indicate the the direction of background field and solid circles indicate the directions of the magnetization. Direction of the magnetization shown in the polar plot (f)-(j). Dotted and Solid arrows indicate the directions of the magnetization before and after current pulses. The PHR values measured 60 s before and after current pulses are shown in the bottom part of the panel (f)-(j).

Table. S3. NOR Logic

| INPUT | | | OUTPUT | |
| --- | --- | --- | --- | --- |
| A | B | Pulse current (µA) | PHR(Ω) | State |
| 0 | 0 | 100 | +26 | 1 |
| 0 | 1 | 250 | -26 | 0 |
| 1 | 0 | 250 | -26 | 0 |
| 1 | 1 | 400 | -26 | 0 |
